# Book Review: The Prevention of Cardiovascular Disease Through the Mediterranean Diet

**DOI:** 10.3389/fphys.2019.00052

**Published:** 2019-02-07

**Authors:** Esther Udo Asamudo, Chidinma Adanna Okolo

**Affiliations:** Department of Physiology, School of Biomedical Sciences, University of Otago, Dunedin, New Zealand

**Keywords:** Mediterranean diet (MD), cardiovascular health, Mediterranean countries, nutrition, cardiovascular disease

This book describes the Mediterranean diet (MD), explored its nutritional constituents and its beneficial health effects. It explains in detail how consumption of MD helps to prevent cardiovascular diseases. It also delineates how much wrong diet contributes to development and progression of heart diseases.

In this book, MD was defined as a healthy eating model, dating back to early 1960s and observed in Greece and Southern Italy. It was also described as a traditional diet obtained from Mediterranean countries, consisting of foods such as grains, vegetables, fruits, olive oil, nuts, fish, dairy products, eggs, meat, herbs, and spices.

The book is made up of 12 chapters which gave in-depth explanation of what MD is. The composition as well as methods with which to achieve and adapt to MD, with the aim of preventing cardiovascular diseases, were highlighted.

The introductory chapter talks about the traditional healthy MD pyramid, the biological and epidemiological evidences regarding the benefits of MD. This chapter discusses the several clinical trials and results involving the adherence to MD together with how it has impacted on the risk of cardiovascular diseases. The authors described the positive results from several clinical trials and mentioned that the significant PREDIMED trial showed ~30% decrease in cardiovascular disease risk factors. They also highlighted the fact that other lifestyle-related factors could be affecting the potential effect of MD on the improvement of cardiovascular health.

Chapters 3 to 8 revealed the importance of major components of MD; fats, oil, mixed nuts, fruits and vegetables, cereals and legumes, fish and meats. Chapter 9 highlights the benefits of moderate consumption of red wine to the body. Chapter 10 lays emphasis on the relationship between high life expectancy rates, low mortality rates and Mediterranean countries with regards to adopting a social lifestyle. Chapter 10 also elaborates on how climate conditions can impact on cardiovascular health and stresses the importance of mental well-being in maintaining a healthy lifestyle. Chapter 11 covers healthy diet and effect of MD on heart and brain function and lists different studies (between 2006 and 2017) which have attempted to show association between MD and improvement of cognitive function.

The 12th Chapter which is the concluding chapter provides details of 30 short and simple recipes containing the different classes of food in MD.

Each chapter contained illustrations, in form of figures and tables highlighting more details about the subject. For instance, the Mediterranean Diet pyramid which showed details of types of foods and the amount to be consumed. This is helpful for readers who understand better with visuals.

In this book, some of the illustrated figures appeared blur due to low image quality. We also observed that the pattern in each chapter was slightly different which could be due to the different contributing authors. However, all chapters included references which would be very helpful for anyone interested in researching more about the Mediterranean Diet.

In summary, this book emphasized the fact that MD is a highly recommended eating model to be adopted toward the prevention of cardiovascular diseases. We do recommend this text for students, physicians, and researchers in areas such as cardiovascular and nutritional sciences. It contains guidelines on dietary patterns that can help improve the health of susceptible and healthy individuals, aiming to maintain a healthy lifestyle.

## Author Contributions

EA and CO read the book and drafted the review summary together. EA typed the manuscript and edited. CO proofread and made final corrections.

### Conflict of Interest Statement

The authors declare that the research was conducted in the absence of any commercial or financial relationships that could be construed as a potential conflict of interest.

